# Review of patient-reported outcomes in multiple myeloma registrational trials: highlighting areas for improvement

**DOI:** 10.1038/s41408-021-00543-y

**Published:** 2021-08-31

**Authors:** Laura L. Fernandes, Jiaxi Zhou, Bindu Kanapuru, Erica Horodniceanu, Thomas Gwise, Paul G. Kluetz, Vishal Bhatnagar

**Affiliations:** 1 Division of Biometrics IX, Center for Drug Evaluation and Research, U.S. Food and Drug Administration, Silver Spring, MD USA; 2 Division of Hematologic Malignancies II, Center for Drug Evaluation and Research, U.S. Food and Drug Administration, Silver Spring, MD USA; 3 Oncology Center of Excellence, U.S. Food and Drug Administration, Silver Spring, MD USA

**Keywords:** Drug development, Quality of life, Myeloma, Myeloma, Biostatistics

## Abstract

Over the past 13 years, there have been advances in characterizing the patient experience in oncology trials, primarily using patient-reported outcomes (PROs). This review aims to provide details on the PRO measures and analyses used in multiple myeloma (MM) registrational trials. We identified registrational trials supporting MM indications from 2007 to 2020 from FDA databases. Trial protocols, statistical analysis plans, and clinical study reports were reviewed for PRO measures used, collection methods, statistical analyses, baseline and instrument completion definitions, and thresholds for clinical meaningfulness. Twenty-five trials supporting 20 MM indications were identified; 17 (68%) contained submitted PRO data. Of the 17 trials, 14 were randomized controlled trials and the remainder were single-arm trials. All but one trial were open label trials. Seven trials collected data electronically and five in paper format. The majority of trials evaluated at least two PRO measures (82%) with two trials (12%) utilizing four measures. Nine unique PRO measures were used, most commonly the EORTC QLQ-30 (87%), EQ-5D (65%), and QLQ-MY20 (47%). All 17 (100%) trials provided descriptive summaries, 10 (59%) carried out longitudinal mixed model analysis, 9 (53%) conducted responder analysis, and 2 (12%) did a basic inferential test. We noted substantial heterogeneity in terms of PRO collection methods, measures, definitions, and analyses, which may hinder the ability to effectively capture and interpret patient experience in future MM clinical trials. Further research is needed to determine the most appropriate approaches for statistical and analytical methodologies for PRO data in MM trials.

## Introduction

The US Food and Drug Administration (FDA) has approved multiple drugs and biologics for the treatment of patients with multiple myeloma (MM) based on clinician-assessed primary endpoints of progression-free survival or overall response over the past 13 years. The US package insert (USPI) of each drug or biologic contains relevant safety and efficacy information from their pivotal trials. There is an increased interest among patients and healthcare providers to understand the patient experience with these novel therapies. One way to measure the patient experience in oncology clinical trials is through the use of patient-reported outcome (PRO) measures [1].

Although PRO information is frequently collected in registrational clinical trials, this information rarely is included in the USPI for many reasons, including lack of flexibility (e.g., space and color limitations) [2]. Analyses of PRO outcomes are rarely included as pre-specified research question or as an endpoint in the statistical testing hierarchical procedure controlled for type 1 error rate [3]. To date, there have been 15 oncology and malignant hematology approvals that resulted in PRO inclusion in the USPI. Although this represents a very small share of oncology and malignant hematology approvals, approximately half (7 of the 15 instances) of PRO in malignant hematology and oncology labeling were for approvals that occurred since 2017, likely due to improvements in the collection and analysis of this type of clinical data [4]. Despite the increase in MM approvals over the past two decades, no MM product labels contain PRO information in the USPI. This review aims to provide an overview of the landscape of which PRO measures are used and how PRO data are collected and analyzed in registrational MM trials. We also aim to provide details on the variety of instruments used, the diversity of the statistical analyses plans in analyzing the data, inconsistencies in data analyses and presentation, and recommendations that may facilitate robust collection and analysis of PRO data in the clinical trials.

Unlike treatment regimens for many oncology and hematological malignancies, particularly in the era of small molecules and immunotherapies, almost all MM therapies are administered as multi-drug combination therapy. In many MM registrational trials, the experimental treatment is added on to an existing backbone therapy giving rise to doublet, triplet, and quadruplet regimens. Given this treatment paradigm, our review also highlights the unique considerations when measuring PRO in add-on trials. Given the growing incorporation of PRO in registrational trials, in part as a result of legislative and regulatory initiatives [5, 6], and the rapid pace of MM approvals over the past 5 years, we conducted a landscape analysis of PRO assessment in MM trials.

## Data collection

### Search strategy and selection criteria

We identified pivotal trials supporting approved MM indications submitted to the FDA between January 2007 and January 2020. LLF, JZ, and VB identified the approvals by querying FDA databases. Similar to Zhou et al. [7] and Fiero et al. [3], marketing applications from the Center for Biologics and the Center for Devices were not included in this review. We excluded PRO instruments that were used specifically for the economic utilization of resources in the submissions since these analyses are typically not reported to the FDA. LLF and JZ reviewed the protocols, statistical analysis plans (SAPs), and clinical study reports (CSRs) for each of the approvals to complete a spreadsheet with different variables of interest as done by Fiero and colleagues [3]. The protocol and the SAP for the trials were reviewed to collect the variables that were pre-specified before the data were analyzed and presented in the CSR. Discrepancies, if any, between interpretations were resolved by discussion among the authors.

### Pre-specified PRO concepts

A PRO concept was considered to be pre-specified if it was defined in the protocol or SAP. The term PRO concept refers to the specific quantity being measured and represents how a patient functions or feels [8]. For example, the European Organisation for Research and Treatment of Cancer Quality of Life Questionnaire—core Questionnaire (EORTC QLQ-C30) is a PRO instrument with 30 items/questions and one of the concepts it measures is physical function. We assessed whether a clear rationale was provided for the choice of PRO concept(s) used in each trial and whether the PRO hypotheses were specific enough to inform the statistical method used for the PRO concept [9]. We also recorded if the PRO concept was analyzed as a domain-level score or an individual item-level score. A domain is a sub-concept in an instrument (e.g., the EORTC QLQ-C30 instrument is comprised of multiple domains, including physical, role, and social function) while an item is an individual question in a particular instrument [8].

The item burden is the sum of the items over all PRO concepts in the trial at each assessment visit [10]. We report summary statistics of the item burden in terms of means and medians at the baseline visit for all of the trials.

### Statistical analyses

In each of the trials, we recorded the various statistical analyses performed for PROs. Within the type of instrument used, the PRO analysis by each PRO concept, at the domain level and single item level were recorded.

The PRO statistical analyses were grouped into four categories, longitudinal analyses, basic inferential tests or general linear models, descriptive summaries, and responder analysis. A responder analysis was defined as an analysis of the proportion of patients who achieved a pre-defined change in score by a certain time point.

We report the description of the PRO analysis population as it might differ from the intent to treat (ITT) or all randomized patient population. We also report on whether a hierarchical testing procedure was followed for controlling the type 1 error rates in multiple hypothesis testing of the PRO concepts. In addition, we recorded the definition used for capturing the baseline measurement, which is used in the change from baseline analysis.

### Instrument completion

Evaluating the completion rate of each PRO instrument is an essential component of the FDA’s examination of PRO data. Generally, PRO completion is defined as the percentage of patients who were still considered on study and had an observed PRO at a specific time point [11]. We recorded whether a PRO instrument completion table was reported in the CSR and the definitions of the numerator and the denominator used for reporting the instrument completion rate.

### Missing data and sensitivity analysis

We recorded if a missing data method was used for the PRO data analysis by use of an imputation method for missing data. We also report if any sensitivity analysis were pre-specified for the missing data and if presented in the CSR.

### Clinical relevance

The thresholds for defining the responder definition might vary by study population [8]. We record if a clinically meaningful threshold was specified and whether a justification in terms of a reference was provided.

## Findings

### Trial characteristics

**Fig. 1:**
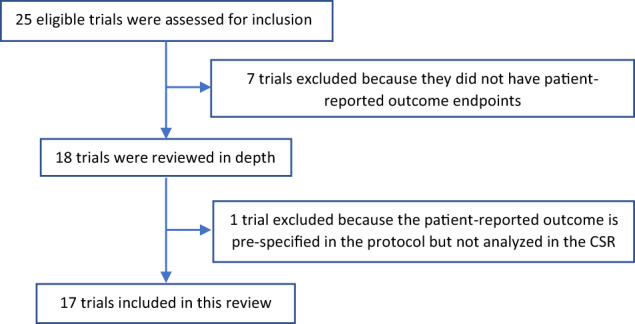
Consort diagram/flowchart for trials analyzed in the review. CSR clinical study report.

We identified 25 clinical trials, supporting 20 indications across 9 distinct products. Of the 25 eligible trials for inclusion, a total of 17 trials were included in this review. Seven trials were excluded because they did not collect PRO data and one trial did not report the analysis in the CSR (consort diagram; Fig. 1).

Three were single-arm trials and 14 were randomized trials. Of the 14 randomized trials, 13 were open label and only 1 was double blinded. Thirty-two treatment regimens were evaluated across the 17 trials either as monotherapy or as combination regimens: 3 (10%) monotherapy, 15 (47%) doublet, 12 (38%) triplet, and 2 (6%) quadruplet therapies. The majority of the trials (*n* = 12) were in the second-line or later setting. Seven trials collected PRO data electronically, five in paper format and five trials did not specify the format in the protocol. The median sample size of the ITT population from the 17 trials was 569 patients (range: 46, 1619).

Across the 17 trials, 40 PRO instruments were used, and 14 trials had ≥2 different PRO instruments included. The most common instruments evaluated were the EORTC-QLQ-C30 (*n* = 13), EQ5D (*n* = 11) and the EORTC-QLQ MM module EORTC-QLQ-MY20 (*n* = 8). Of the 11 trials with EQ5D, 7 used the EQ-5D-5L and 4 used EQ-5D-3L (Table 1).

**Table 1:** Summary characteristics of the pivotal trials used for MM approvals in the US.

	Trials (*n* = 17)
Study design	
Randomized	14 (82%)
Single arm	3 (18%)
Number of therapies (*n* = 32)	
Monotherapy	3 (10%)^a^
Doublet	15 (47%)^a^
Triplet	12 (38%)^a^
Quadruplet	2 (6%)^a^
Blinding status	
Blinded	1 (6%)
Open label	16 (94%)
Line of therapy	
First	3 (18%)
Second line and beyond	12 (70%)
Maintenance	2 (12%)
Number of PRO instruments	
1	3 (18%)
2	7 (41%)
3	5 (29%)
4	2 (12%)
Collection format	
Electronic	7 (42%)
Paper	5 (29%)
Not specified	5 (29%)
Type of instrument name	
EORTC QLQ-C30	13 (76%)
EORTC QLQ-MY20	8 (47%)
MDASI-MM	1 (6%)
BPI-SF	2 (12%)
EQ-5D—5L	7 (41%)
EQ-5D—3L	4 (23%)
FACT/GOG-NTX	3 (18%)
MRU	1 (6%)
FACT-MM	1 (6%)
Item burden	
Mean (SD)	48 (13)
Median (range)	42 (32, 75)

*EORTC QLQ-C30* European Organization for Research and Treatment of Cancer Quality of Life Questionnaire—core Questionnaire, *EORTC QLQ-MY20* EORTC QLQ—Multiple Myeloma Module, *MDASI-MM* MD Anderson Symptom Inventory—Multiple Myeloma Module, *BPI-SF* Brief Pain Inventory- Short Form, *EQ-5D-5L* EQ-5D-5 Levels, *EQ-5D-3L* EQ5D-3 Levels, *FACT/GOG-NTX* Functional Assessment of Cancer Treatment Gynecologic Oncology Group—Neurotoxicity, *MRU* Medical Resource Utilization, *FACT-MM* Functional Assessment of Cancer Treatment—Multiple Myeloma.

^a^Percentage based on the total number of therapies.

Timing of assessments in the trials were heterogeneous and usually sparse (e.g., every 3 months). In all but one trial, the experimental drug was given early within a cycle, while the backbone therapy was continued through the majority or all of the cycle. Optimal timing of PRO assessment for experimental MM combination regimens in comparative trials will be discussed in a future manuscript.

### PRO concepts

All of the 17 (100%) trials pre-specified a PRO concept in the protocol as at least an exploratory endpoint or outcome, but a pre-specified concept rationale was provided in only one instance. Twenty-four out of 40 (60%) instruments were included as exploratory endpoints, while 16 (40%) were evaluated as secondary endpoints. The burden at baseline was a mean of 48 items (SD = 13) with a median of 42 items (range: 32,75).

### Statistical methods

All of the 17 (100%) trials provided descriptive summaries, 10 (59%) carried out longitudinal mixed model analysis, 9 (53%) performed a responder analysis, and 2 (12%) performed a basic inferential test. Most of the trials carried out domain- and item-level analyses (Table 2).

**Table 2:** Statistical analysis of pre-specified PRO in the SAP concepts, domain, and single items of the 17 trials reviewed.

	Studies with pre-specified PRO concepts (*N* = 17)	Instrument with domain-level analyses (*N* = 40)	Instrument with item-level analyses (*N* = 40)
Longitudinal mixed model analysis	10 (59%)	25 (63%)	8 (20%)
Basic inferential test or general linear model	2 (12%)	6 (35%)	0
By time point	2 (12%)	6 (35%)	0
1-sample and 2-sample *t* test	2 (12%)	6 (35%)	0
Descriptive summaries	15 (88%)	37 (93%)	37 (93%)
Summary statistics	15 (88%)	37 (93%)	37 (93%)
Change from baseline	15 (88%)	37 (93%)	37 (93%)
Paired/un-paired *t* test	1 (6%)	3 (8%)	0
Cumulative distribution function figures	1 (6%)	2 (5%)	0
Responder analysis	9 (53%)	21 (53%)	7 (18%)
Cumulative distribution function figures	3 (18 %)	7 (18%)	7 (18%)
Stratified CMH test	2 (12%)	2 (5%)	0
Stratified Cox regression	5 (29%)	9 (23%)	0
Unstratified log-rank test	2 (12%)	4 (10%)	0
Kaplan–Meier estimate	4 (24%)	8 (20%)	0

A change from baseline analysis was evaluated in all 17 trials but pre-specified in only 15 (88%) trials either using descriptive summary statistics or by a longitudinal mixed modeling approach in 10 (59%) trials. There was variability in the details of the mixed models specified, ranging from specifying no details, general mention that “a mixed effects model will be used to analyze the change in baseline measurements,” to specifying the covariates used as fixed and/or random and including details of the covariance matrix structure to account for the correlations across multiple cycles. There were differences in terms of the covariates used in the model and which variables were treated as fixed or random. All models were fitted to provide estimates for the change from baseline but estimated different quantities when taking the covariates adjusted, the covariance matrix, and the random and fixed terms into consideration.

A variety of methods were used for the responder analysis. The stratified Cox Regression (*n* = 5) and Kaplan–Meier estimate (*n* = 4) in a time-to-event analysis were most commonly used. Specifications of the censoring rules for the analysis differed across trials. Based on our categories of statistical analysis, trials most frequently did 2 (range: 2–7) statistical analyses for a single PRO instrument. None of the trials included multiplicity type I error controls for PRO analyses. None of the PRO analyses were included in USPIs.

#### PRO population

There were differences observed in the definition of the PRO analysis population. Seven (41%) included all patients in the ITT population and 2 (12%) included an additional condition of a post-baseline measurement or a baseline assessment, while 2 (12%) included patients in the safety population defined as the ITT population receiving the study drug. The remaining 6 (35%) of the trials were variations on the safety and ITT populations, presented in Table 3.

**Table 3:** PRO patient population included in the PRO analyses.

PRO evaluable population definition	Trials (*N* = 17), *n* (%)
Safety population with baseline and at least one post-baseline assessment	1 (6)
Safety population	2 (12)
ITT patients with baseline and at least one post-baseline assessment	2 (12)
ITT	7 (41)
Patients with at least one post-baseline assessment and 50% completion of the relevant items for a domain	2 (12)
At least one assessment	2 (12)
ITT patients receiving active treatment and at least one PRO measurement item completed	1 (6)

*ITT* intent to treat.

#### Definition of baseline

Differences in baseline definition were observed in terms of collection and assignment of baseline visit. Seven trials (41%) defined baseline to be “Cycle 1, Day 1” measurements, two trials (12%) defined baseline measurements to be “on or prior to Cycle 1, Day 1”, and 8 (47%) of the trials defined as “screening phase/before randomization.”

#### Definition of instrument completion

Definition of compliance of the PRO measures varied across the trials. One trial (6%) defined compliance as “completing all questions,” two trials (12%) defined as “completing 50% of the questions,” and the remaining 14 trials (82%) as “completing enough items to calculate the score in any domain” or some variant, captured in Table 4.

**Table 4:** Definition of Compliance.

Definition numerator/denominator	Trials (*N* = 17), *n* (%)
Completed all questions/expected	1 (6)
Completed 50% of all questions in the instrument (all in the case of EQ-5D)/expected	2 (12)
Completed questions in at least one of the subscales/expected	1 (6)
Completed at least one questionnaire/expected	2 (12)
Forms actually completed/expected	1 (6)
Unclear/unclear	1 (6)
Unclear/expected	9 (53)

*EQ-5D-5L* EQ-5D-5 Levels.

### Missing data and sensitivity analysis

Imputation of missing data using last observation carried forward was specified in the SAP for only one of the trials. Three trials specified sensitivity analysis for missing PRO data in terms of a random slope, pattern mixture model, and use of an ancillary variable. Two out of the three trials presented the pre-specified sensitivity analysis results in the CSR.

#### Clinical thresholds

We observed variability in the definition and justification of clinical thresholds across trials despite use of the same instrument and similar MM populations, captured in Table 5. The minimal important difference (MID) for global health status specified 8 and 5 points in 2 and 3 trials, respectively, using Kvam and colleagues [12], Cocks and colleagues [13], or Delforge and colleagues [14] as the justification. Bedard and colleagues [15] was used to justify a MID of 15.7 points and Cocks and colleagues [13] for 5 points for pain in 2 and 1 of the trials, respectively. EORTC QLQ-MY20 domain scores for side effects were justified by using Dimopoulos and colleagues [16] for MID of −6 and −3 points, while MID of 7 points was estimated within the study using Cronbach’s alpha. In the case of disease symptoms, Dimopoulos and colleagues [15] was used to justify MID of −10 and −5 points, while MID of 9 points was estimated within the study. The BPI-SF instrument was used in two trials but had different definitions for response. In one trial, a 30% reduction in worst pain along with no increase in analgesics was considered a response but no justification in terms of a reference was provided. The second trial used McQuellon and colleagues [17], a cervical cancer reference, to justify a score of 6 (on a scale of 0–10) as a responder.

**Table 5:** Thresholds for minimal important difference (MID).

	MID	Number (*N*)
EORTC QLQ-C30 (*N* = 13)		
GHS	8	2
	5	3
Pain	15.7	2
	>6 points	1
EORTC QLQ-MY20 (*N* = 8)		
Side effects	−6	1
	−3	1
	7	1
Disease symptoms	−10	1
	−5	1
	9	1
BPI-SF (*n* = 2)		
	30% reduction in worst pain; no increase in analgesics	1
	A score of 6 on a scale of 0–10 on any single item	1

*EORTC QLQ30* European Organization for Research and Treatment of Cancer Quality of Life Questionnaire—core Questionnaire, *EORTC QLQ-MY20* EORTC QLQ—Multiple Myeloma Module, *BPI-SF* Brief Pain Inventory—Short Form.

## Discussion

Our review of the collection and analysis of the PRO data in pivotal MM trials for drug approvals between January 2007 and January 2020 identified substantial heterogeneity with respect to PRO collection methods, definitions of analysis population, instrument completion, and clinical meaningful changes. We also found substantially different methods for the handling of missing data and the use of statistical methods for analyses. These differences in PRO analyses within the same disease and therapeutic setting may hinder the ability to effectively capture and interpret patient experience in MM clinical trials, which are valuable information for patients and clinicians.

There was also substantial heterogeneity between trials in terms of the definitions of baseline assessment and analysis populations, as well as the choice of analysis methods. As per the ICH E9 addendum [18], the estimand framework calls for the proper definition of quantities of interest. Fiero and colleagues [19] describe the five attributes of the estimand framework for PRO in oncology stressing the need for defining the treatment, the study population, the endpoint, the intercurrent events, and the summary statistic. Consistent definition of baseline and the imputation of baseline from screening visits should be specified, ideally prior to trial initiation. For the change from baseline analysis, calculation of the PRO score, handling of missing items, and the analysis model need to be well defined. Specifically, handling of missing patients due to adverse events, hospitalization, or death should be clearly addressed; however, in our review, we did not find this to be a commonplace practice. It was concerning to note that only three trials specified sensitivity analyses for missing PRO data, but as recommended by multiple statisticians, it is essential to include these SAPs, ideally prior to trial initiation [20, 21]. Missing data occurs frequently in clinical trials due to treatment discontinuation, toxicity, and tumor progression [22–24]; however, adequate measures need to be adopted to avoid biased results due to a reduction in power owing to missing data [25]. Demonstrating a robust treatment effect through the use of sensitivity analyses increases the confidence in the observed estimates.

Although change from baseline analysis was done in a large proportion of trials we examined, the statistical models used in all of the trials were different despite similarities in the patient populations, designs of the trials, and treatments being studied. Differences in adjustments for the covariates and specification of the variance across the visits varied in all of the models. All models provide estimates for the change from baseline but estimate different quantities when taking the model specifications into account. Assessing how an endpoint changes over the duration of the trial requires defining the baseline value for that endpoint. We noted substantial variability in defining, identifying, and collecting baseline measurements in our review, which could affect the interpretability of the change from baseline analyses and endpoints focused on demonstrating improvement/deterioration.

We found differences between the analyses pre-specified in the SAP and those actually reported in the corresponding CSR. In one example, we noted significant differences in how the change from baseline analysis was specified in both documents. In another example, time to deterioration of global quality of life was not specified in the protocol but reported in the CSR. Pre-specifying the PRO objectives in the protocol and SAP helps in selecting the appropriate analyses method [1] and in choosing a proper plan to control for type 1 error for multiplicity in hypothesis testing [26].

Time-to-event endpoints are based on a responder definition, which differed across the trials depending on the definition of MID and the domains used within the same instrument. Justifications for selecting the thresholds were not provided in all of the trials and varied thresholds were noted for the same PRO measure across different trials. This difference coupled with the varied censoring rules adds another layer of heterogeneity in analysis of the endpoint. Definitions of thresholds should be selected based on the relevant population being studied and discussed with regulatory bodies [5].

We generally note that, across the trials, patients were asked to complete a range of 32–75 PRO items. We have long known that high completion rates can be attained even with item burden as high as 60–80; however, we continue to advocate for the least number of items that can address stated study objectives. FDA’s recently published core outcome draft guidance [10] outlines ways that assessment of symptoms and function could be achieved while still aiming to minimize patient burden.

There are several limitations of our review of PRO in registrational MM trials. Our findings do not represent all clinical trials across oncology and malignant hematology. In addition, MM marketing applications submitted to the Center for Biologics and the Center for Devices were excluded in our review. We recognize that our findings may not be generalizable to trials in other malignant hematologic diseases, other cancer trials that are not intended to support a marketing application (i.e., early-phase trials), and trials in non-oncology populations.

Our review has several unique strengths. Most previous reviews are based on publications or protocols [27], but we examined and reviewed all the pivotal trial protocols, CSRs, and SAPs in the FDA database for PRO descriptors. While Fiero and colleagues [3] conducted a similar review of PRO endpoints in lung cancer trials, to our knowledge, this is the first review in MM focusing on PRO. Although we have observed substantial heterogeneity for PRO collection in MM clinical trials, we are hopeful that these methods will improve as Sponsors adopt best practices. Sustained effort by the FDA review teams to work with sponsors during the trial design phase have improved consistency in concepts, instruments, assessment frequency, and pre-specification of PRO objectives. Higher-quality data submissions have helped the FDA to communicate PRO data across multiple media, including FDA labels, review summaries, and Project Patient Voice [2]. Project Patient Voice [2] is a FDA Oncology Center of Excellence pilot in which high-quality PRO data from cancer registrational trials can be communicated to the public. FDA has described a core outcome set to be included in cancer clinical trials, which includes assessment of patient-reported adverse events, disease symptoms, physical function, role function, and overall side effect bother [28]. Use of this core outcome set in future MM registration trials is one step toward consistency in PRO collection and analysis. Alignment of PRO research questions with the estimand framework, clear description of statistical methods, and justification for thresholds will lead to more meaningful PRO results that can be shared with patients and healthcare providers. FDA is participating in the Setting International Standards in Analyzing Patient-Reported Outcomes and Quality of Life Endpoints Data consortium in an effort to further improve consistency in analytic methods. To that end, further research is needed to determine the most appropriate approaches for statistical and analytical methodologies for PRO data in MM trials.

## Supplementary information

Checklist
